# The complete mitochondrial genome of the Indian leafwing butterfly *Kallima paralekta* (insecta: Lepidoptera: Nymphalidae)

**DOI:** 10.1080/23802359.2020.1862000

**Published:** 2021-01-27

**Authors:** Cassidy P. Aguila, Ryan M. Aikens, Parneet K. Ateliey, Hannah M. Buhr, Michael G. Castro, Rayeil J. Chua, Nishtha Dayal, Heather N. Deane, Brendan Dennehy, Meerim Esenbekova, Jessica L. Fay, Carly Gair, Brady R. Gordon, Soomin Huh, Fariba Ishrar, Elizabeth B. Jonson, Charanpreet F. Kaur, Clémence Kokolo, Katrina Lanyon, David Laudato, Tri Q. Le, McKay Lowry, Imane Marrakchi, Ruth Marte, Connor S. McIntyre, Jaime C. McNicholl, Gabrielle B. Nowlin, Claudia Pfeifer, Luc J. Posillipo, Shamsa Ricci, Sean M. Robertson, Jillian Roziere, Prerna Sharma, Danilo Shevkoplyas, Holly J. Stokes, Rebecca E. Twilley, Chenyi Wang, Jennifer K. Watt, Arizona G. Wilkinson, Jenelle M. Williams, Michael D. Wood, Heeeun Yang, Jeffrey M. Marcus

**Affiliations:** Department of Biological Sciences, University of Manitoba, Winnipeg, Canada

**Keywords:** Illumina sequencing, leaf-mimicry, Lepidoptera, masquerade, mitogenomics

## Abstract

The Indian leafwing butterfly *Kallima paralekta* (Horsfield, 1829) (Nymphalidae) is an Asian forest-dwelling, leaf-mimic. Genome skimming by Illumina sequencing permitted assembly of a complete circular mitogenome of 15,200 bp from *K. paralekta* consisting of 79.5% AT nucleotides, 22 tRNAs, 13 protein-coding genes, two rRNAs and a control region in the typical butterfly gene order. *Kallima paralekta COX1* features an atypical CGA start codon, while *ATP6, COX1, COX2*, *ND4*, *ND4L,* and *ND5* exhibit incomplete stop codons completed by 3’ A residues added to the mRNA. Phylogenetic reconstruction places *K. paraleckta* within the monophyletic genus *Kallima*, sister to *Mallika* in the subfamily Nymphalinae. These data support the monophyly of tribe Kallimini and contribute to the evolutionary systematics of the Nymphalidae.

The Living Prairie Mitogenomics Consortium is an undergraduate structured inquiry exercise (Marcus et al. 2010) assembling arthropod mitogenomes for improved DNA-based species identification and phylogenetics (Living Prairie Mitogenomics Consortium 2017, 2018, 2019, 2020; Marcus 2018). Student participants analyzed sequence data (further curated by the instructor) for presentation here.

Alfred Russell Wallace (1867) described the close resemblance between the appearance of Indian leafwing butterfly *Kallima paralekta* (Nymphalidae) and dead leaves with respect to shape, size, background coloration, markings, and perching behavior as ‘the most wonderful and undoubted case of protective resemblance in a butterfly which we have ever seen’. Now often referred to as masquerade mimicry (Skelhorn 2015), the origin and evolution of this protective resemblance has been an important case study in the discussion of how organisms produce complex phenotypes (Suzuki et al. 2014). Convergent evolution of leaf masquerade mimicry in nymphalid butterfly lineages has also been a challenge for taxonomists interested in *Kallima* (Shirôzu and Nakanishi 1984; Larsen 1991) and phenotypically similar species in genera such as *Kallimoides* (Payment et al. 2020a), *Mallika* (Alexiuk et al. 2020b), *Doleschallia* (Hamilton et al. 2020), and *Junonia* (Wahlberg et al. 2005).

To facilitate future phylogenetic and taxonomic work, here we report the complete mitochondrial genome sequence of *K. paralekta* from specimen Kpar2017.1, collected in Pattaya, Thailand (GPS 12.927608 N, 100.877083 E) in 2017, that has been pinned, spread, and deposited in the Wallis Roughley Museum of Entomology, University of Manitoba (voucher WRME0507735).

DNA was prepared from a specimen leg using a DNeasy Blood and Tissue kit **(**Qiagen, Düsseldorf, Germany) with slight modifications to the standard protocol as described in McCullagh and Marcus (2015). DNA was sheared by sonication and a fragment library was prepared using the NEBNext Ultra II DNA Library Prep Kit for Illumina (New England Biolabs, Ipswich, Massachusetts) as previously described (Peters and Marcus 2017), before sequencing by Illumina NovaSeq6000 (San Diego, California) (Marcus 2018). Mitogenome assembly of *K. paralekta* (Genbank accession MW192438) was performed by mapping the resulting sequence library of 23,143,566 paired 150 bp reads (Genbank SRA PRJNA667707) to a *K. inachus* reference mitogenome (HM243591) using 5 iterations of the medium sensitivity settings of Geneious Prime 2020.2. Annotation was in reference to *K inachus* and *Junonia stygia* (Nymphalidae, MN623383 (Living Prairie Mitogenomics Consortium 2020)). The *K. paralekta* nuclear rRNA repeat (MW192439) was also assembled and annotated using *J. stygia* (MF680448 (Living Prairie Mitogenomics Consortium 2020)), *Anartia jatrophae* (MT742579 (Payment et al. 2020b)), and *Araschnia levana* (MT750296 (Alexiuk et al. 2020a)) reference sequences.

The *K. paralekta* circular 15,200 bp mitogenome assembly was composed of 40,332 paired reads with nucleotide composition: 39.7% A, 12.7% C, 7.8% G, and 39.8% T. The gene order and composition of the *K. paraleckta* mitogenome is identical to all known butterfly mitogenomes (Cao et al. 2012; McCullagh and Marcus 2015; McCullagh et al. 2020).

Ten *K. paralekta* mitochondrial protein-coding genes begin with typical ATG or ATT start codons, with the remaining genes beginning with ATC (*ND6*), GTG (*COX2*), or CGA (*COX1*) start codons (Liao et al. 2010). The mitogenome contains three protein-coding genes (*COX1, COX2, ND4L*) with single-nucleotide (T) stop codons, and three protein-coding genes (*ATP6, ND4, ND5*) with two-nucleotide (TA) stop codons completed by post-transcriptional addition of 3′ A residues. The locations and structures of tRNAs were determined using ARWEN v.1.2 (Laslett and Canback 2008). The tRNAs have typical cloverleaf secondary structures except for trnS (AGN) where the dihydrouridine arm is replaced by a loop, while the mitochondrial rRNAs and control region are typical for Lepidoptera (McCullagh and Marcus 2015).

We reconstructed a phylogeny using the complete *K. paralekta* mitogenome, three *K. inachus* mitogenomes (Qin et al. 2012; Liu et al. 2020), and 30 additional mitogenomes from family Nymphalidae, including *Melitaea cinxia* (HM243592) and *Mellicta ambigua* (MK252271) as outgroup species. Mitogenome sequences were aligned in CLUSTAL Omega (Sievers et al. 2011) and analyzed by parsimony and maximum likelihood (model selected by jModeltest 2.1.7 (Darriba et al. 2012) and likelihood ratio test (Huelsenbeck and Rannala 1997)) in PAUP* 4.0b8/4.0d78 (Swofford 2002) (Figure 1). Phylogenetic analysis places *K. paralekta* within a monophyletic genus *Kallima,* which in turn is sister to another leaf-mimicking genus, *Mallika*. The leaf-mimics in genera *Doleschallia, Kallimoides,* and *Junonia* are only distantly related to *Kallima* and to each other, as has been suggested in some other phylogenetic analyses (Wahlberg et al. 2005; Alexiuk et al. 2020b).

**Figure 1.**
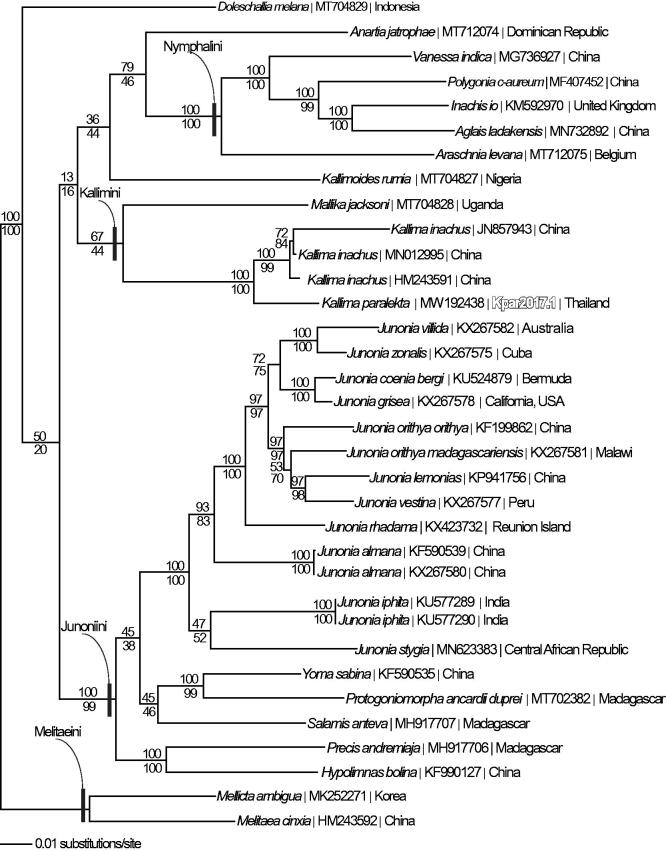
Maximum likelihood phylogeny (GTR + G model, G = 0.2300, likelihood score 116120.69888) of *Kallima paralekta* and 33 additional mitogenomes from subfamily Nymphalinae based on 1 million random addition heuristic search replicates (with tree bisection and reconnection). One million maximum parsimony heuristic search replicates produced an identical tree topology (parsimony score 20514 steps). Numbers above each node are maximum likelihood bootstrap values and numbers below each node are maximum parsimony bootstrap values (each from 1 million random fast addition search replicates).

## Data Availability

The data that support the findings of this study are openly available in GenBank of NCBI at https://www.ncbi.nlm.nih.gov, reference numbers PRJNA667707, MW192438, and MW192439.
